# Japanese encephalitis virus degrades TRAF3 to suppress type I interferon and promote viral replication through NS5 and host TUFM proteins

**DOI:** 10.1186/s13567-026-01740-y

**Published:** 2026-04-04

**Authors:** Xingya Wang, Chen Wang, Xinyu Yang, Wenzhen Qin, Xuelan Liu, Hai Yu, Wu Tong, Guangzhi Tong, Tongling Shan, Ning Kong, Guangxu Xing, Hao Zheng

**Affiliations:** 1 Shanghai Veterinary Research Institute, Chinese Academy of Agricultural Sciences, Shanghai, 200241 China; 2 Jiangsu Co-innovation Center for Prevention and Control of Important Animal Infectious Diseases and Zoonoses, Yangzhou University, Yangzhou, China; 3 Animal-Derived Food Safety Innovation Team, Anhui Agricultural University, Hefei, China; 4 Institute for Animal Health, Henan Academy of Agricultural Sciences, Key Laboratory of Animal Immunology of the Ministry of Agriculture, Zhengzhou, China

**Keywords:** JEV, TUFM, autophagy, p62, degradation, immune escape

## Abstract

Japanese encephalitis (JE), caused by the Japanese encephalitis virus (JEV), remains a major global health concern. A deeper understanding of how JEV manipulates host antiviral responses is critical for developing more effective therapeutic strategies. In this study, we identified a novel immune evasion mechanism through which JEV suppresses host innate immunity to facilitate viral replication. Specifically, JEV infection induces the expression of the host translation elongation factor TUFM by activating the transcription factor JUN. We demonstrated that TUFM promotes JEV replication, while TUFM silencing significantly reduces viral load. Mechanistically, TUFM acts as a scaffold to recruit the E3 ubiquitin ligase Parkin and the autophagy receptor p62, thereby mediating selective autophagy-dependent degradation of the key innate immune adaptor TRAF3. Intriguingly, the viral NS5 protein was also found to promote TRAF3 degradation via a similar autophagy-dependent pathway, suggesting a coordinated strategy between viral and host factors. Since TRAF3 is essential for the activation of IRF3 and downstream type I interferon signaling, its degradation effectively blunts antiviral innate immune responses. Collectively, our findings reveal a previously unrecognized mechanism by which JEV subverts host innate immunity through the TUFM-NS5-Parkin-p62-TRAF3 axis to promote immune evasion and viral propagation.

**Supplementary Information:**

The online version contains supplementary material available at 10.1186/s13567-026-01740-y.

## Introduction

Japanese encephalitis (JE) is caused by the Japanese encephalitis virus (JEV), which is a neurotropic, single-stranded RNA virus belonging to the Flavivirus. JEV can cause reproductive disorders in pigs and encephalitis in newborn piglets, which results in great economic losses in the swine industry [1, 2]. JEV infection results in a broad spectrum of clinical manifestations, from mild febrile illness to severe neurological sequelae such as altered consciousness, motor abnormalities, and seizures. This makes JEV a significant threat to global health [3, 4]. Approximately 68 000 cases are reported annually, with a case fatality rate of 20–30% among those who develop Japanese encephalitis (JE), and 30–50% of survivors suffer from permanent neurological or psychiatric sequelae [5, 6]. This highlights that JEV is a significant virus affecting both public health and the livestock industry.

The JEV genome is translated into a polyprotein that is cleaved to generate three structural proteins—capsid (C), membrane (prM/M), and envelope (E), and seven non-structural proteins (NS1, NS2A, NS2B, NS3, NS4A, NS4B, NS5) [7]. Among these, JEV NS5 has the highest molecular weight and is composed of N-terminal methyltransferase (MTase) and RNA-dependent RNA polymerase (RdRp) domains [8]. JEV NS5 is implicated mainly in the host interferon regulation [9, 10]. Through interplays with the nuclear transporters KPNA3 and KPNA4, the JEV NS5 protein represses the type I IFN triggering, thus inhibiting the IRF3 and NF-κB translocation to nuclei [11, 12]. Nevertheless, the specific mechanism by which NS5 utilizes host proteins to facilitate the immune escape of JEV have not been fully elucidated.

Tu Translation Elongation Factor (TUFM) is a host mitochondrial membrane protein reportedly involved in mitochondrial autophagy, apoptosis, and oxidative stress [13, 14]. A recent report demonstrated that increased levels of TUFM-mediated autophagy are linked to the inhibitory action on the replication of avian signature influenza virus [15]. The RSV NS1, as a novel receptor protein, may interact with TUFM to ultimately result in TUFM-dependent mitochondrial autophagy and IFN-β inhibition [16, 17]. These findings provide new insight into the function of the mitochondrial protein TUFM, which may have important implications for the development of novel antiviral strategies.

Upon infection with RNA viruses, host antiviral immunity is elicited by such pattern recognition receptors (PRRs) as retinoic acid-inducible gene-1-like receptors, NOD-like and Toll-like receptors, and several intracellular nucleic acid sensors [18]. After the recognition of pathogen-related molecular patterns produced by an RNA virus, the IFN signaling pathway is sequentially initiated by PRRs to resist the invasion of the virus. As a family of adaptor molecules present in cells, tumor necrosis factor receptor-associated factors (TRAFs) are an essential constituent of inherent immunity [19]. Eukaryotic cells contain seven types of TRAF proteins (TRAF1–TRAF7) [20], and most of the literature has emphasized the role of TRAF3 as an adaptor molecule in regulating inherent host immunity. It has been reported that RBM14 can enhance host antiviral defense by interacting with TRAF3 to induce IFN expression [21]. PGAM5 initiates the IFN pathway by interacting with TRAF3 to suppress PEDV replication [22]. We demonstrate that the JEV NS5 protein cooperates with the host factor TUFM to degrade the key immune adaptor TRAF3. This degradation inhibits the type I interferon (IFN-I) response, ultimately facilitating enhanced viral replication.

## Materials and methods

### Antibodies and reagents

Mouse monoclonal antibodies against TUFM (67,802–1-Ig) and GAPDH (60,004–1-lg), as well as horseradish peroxidase (HRP)-conjugated anti-mouse (SA00001-1) and anti-rabbit (SA00001-2-Ig) antibodies, were obtained from Proteintech. The anti-HA-tag antibody (3724 T) anti-pIRF3-antibody (#79945 s) was supplied by Cell Signaling Technology, while the mouse anti-Flag-tag antibody (F1804) was a Sigma-Aldrich product. DAPI were procured from Beyotime Biotechnology.

### Cells and viruses

Dulbecco’s modified Eagle medium (Invitrogen, 12430054) containing 10% fetal bovine serum (Gibco, 10099141) was used for the maintenance of human embryonic kidney (HEK293T) cells (ATCC, CRL-11268), whereas Eagle’s minimum essential medium (SUNNCELL, SNM-009B) was utilized to maintain baby Syrian kidney BHK-21 cells (ATCC, CCL-10). We cultured these cells under 37 °C and 5% CO_2_ conditions. JEV were preserved at the Shanghai Veterinary Institute, Chinese Academy of Agricultural Sciences, was used in this study. BHK-21 cells were used for JEV infection and titration, and the titers were determined using Karber’s formula.

### Transfection

After the cells were cultured to about 80%–90% confluence, Lipofectamine 3000 reagent (Invitrogen, L3000015) was applied for the recombinant plasmid transfection into the cells. In the wells of 12-well plates, HEK293T cells at 60%–70% density were transfected with siRNAs using Lipofectamine RNAiMAX (Invitrogen 13778150).

### JEV infection

The JEV genotype I HEN0701 strain was isolated and preserved in our laboratory. In order to establish JEV challenge, BHK-21 cells were inoculated in culture plates until 90% adherence, followed by virus infection at an MOI of 1 at 37 °C for 1 h. Subsequently, the cells were washed with phosphate-buffered saline (PBS) (Gibco, C20012500BT) and cultured again in a freshly prepared medium. Viral titers were determined using Karber’s formula, which refers to reaching 50% of the tissue culture infectious dose (TCID_50_).

### Western blotting

The cells were digested using the RIPA lysis and extraction buffer (89901, Thermo Fisher Scientific) containing a protease inhibitor cocktail (Bimake, B14001). Then, the cellular lysates were heated in 5 × SDS loading buffer, subjected to the SDS-PAGE separation process, and transferred onto nitrocellulose membranes (GE Healthcare, 10600001). After the membrane incubation using primary and secondary antibodies, an enhanced chemiluminescence substrate (Share-bio, SB-WB012) was used for protein quantification.

### Coimmunoprecipitation (Co-IP) assay

After co-transfection of the indicated plasmids for 24 h, NP40 lysis buffer (FNN0021) supplemented with protease inhibitors was used for digesting the cells. The resulting lysates were incubated overnight at 4 °C with anti-Flag-antibody-coupled Dynabeads Protein G (10004D) (both Life Technologies) was utilized for probing the cells. The beads were then washed three times with 0.02% PBST to remove nonspecific bindings. Finally, pH 2.8 glycine buffer (50 mM) was applied to elute the magnetic beads. Co-precipitated proteins were identified through immunoblotting with the designated antibodies.

### GST affinity-isolation assay

After the indicated genes were cloned and inserted into the prokaryotic expression vector pCold-GST (3372, Clontech Laboratories, Inc.) or pCold-TF-Flag (3365, Clontech Laboratories, Inc.), we expressed the genes in competent BL21 cells (C504–03, Vazyme Biotech). The GST Protein Interaction Pull-Down Kit (Thermo Fisher Scientific, 21,516) was subsequently utilized to characterize protein–protein interactions, followed by western blotting of the samples.

### Confocal immunofluorescence assay

HeLa cells were cultured in 6-well plates and transfected with the designated plasmids. At 24 h post-transfection, cells were immobilizated in 4% paraformaldehyde (P6148) and permeabilizated by 0.1% Triton X-100 (T9284) (both Sigma-Aldrich), After blocking with 5% bovine serum albumin (9998, Cell Signaling Technology), the transfected cells were probed using primary and secondary antibodies for 1 h at 37 °C in the dark respectively. Thereafter, the cell nuclei were DAPI stained, followed by antibody fluorescence detection using laser scanning confocal microscopy (Carl Zeiss, Oberkochen, Germany).

### Luciferase reporter assay

HEK293T cells were seeded into 24-well plates at an appropriate density and allowed to adhere overnight. The following day, cells were transfected with the indicated firefly luciferase reporter plasmids and control plasmid (phRL-TK) using Lipofectamine 3000 (Thermo Fisher Scientific). After 24 h of incubation at 37 °C with 5% CO_2_, cells were lysed using 100 μL of passive lysis buffer provided in the Dual-Luciferase^®^ Reporter Assay Kit (DL101, Vazyme Biotech). Luciferase activity was measured using a microplate luminometer.

### iTRAQ

SH‑SY5Y cells were seeded into 6-well plates at an appropriate density and allowed to adhere overnight. The following day, cells were transfected with the pCMV-HA-NS5 plasmids and control plasmid for 24 h. NP40 lysis buffer supplemented with protease inhibitors was used for digesting the cells. The resulting lysates were incubated overnight at 4 °C with anti-HA-antibody-coupled Dynabeads Protein G was utilized for probing the cells. The beads were then washed three times with 0.02% PBST to remove nonspecific bindings. Finally, pH 2.8 glycine buffer (50 mM) was applied to elute the magnetic beads. Co-precipitated proteins were identified through iTRAQ. The results are provided in the Additional file 1.

### Statistical analysis

Image J software was used to calculate the band intensities of the western blots. Data are presented as the mean ± SD from three independent experiments. Pairwise comparisons were made using a two-tailed Student’s t-test. Multiple comparisons were performed with an ANOVA followed by Dunnett’s multiple comparisons test. Significance was evaluated at the *P* < 0.05, *P* < 0.01, and *P* < 0.001 levels, while ns stood for not significant.

## Results

### JEV infection upregulated the expression of TUFM through the transcription factor JUN

**Figure 1:**
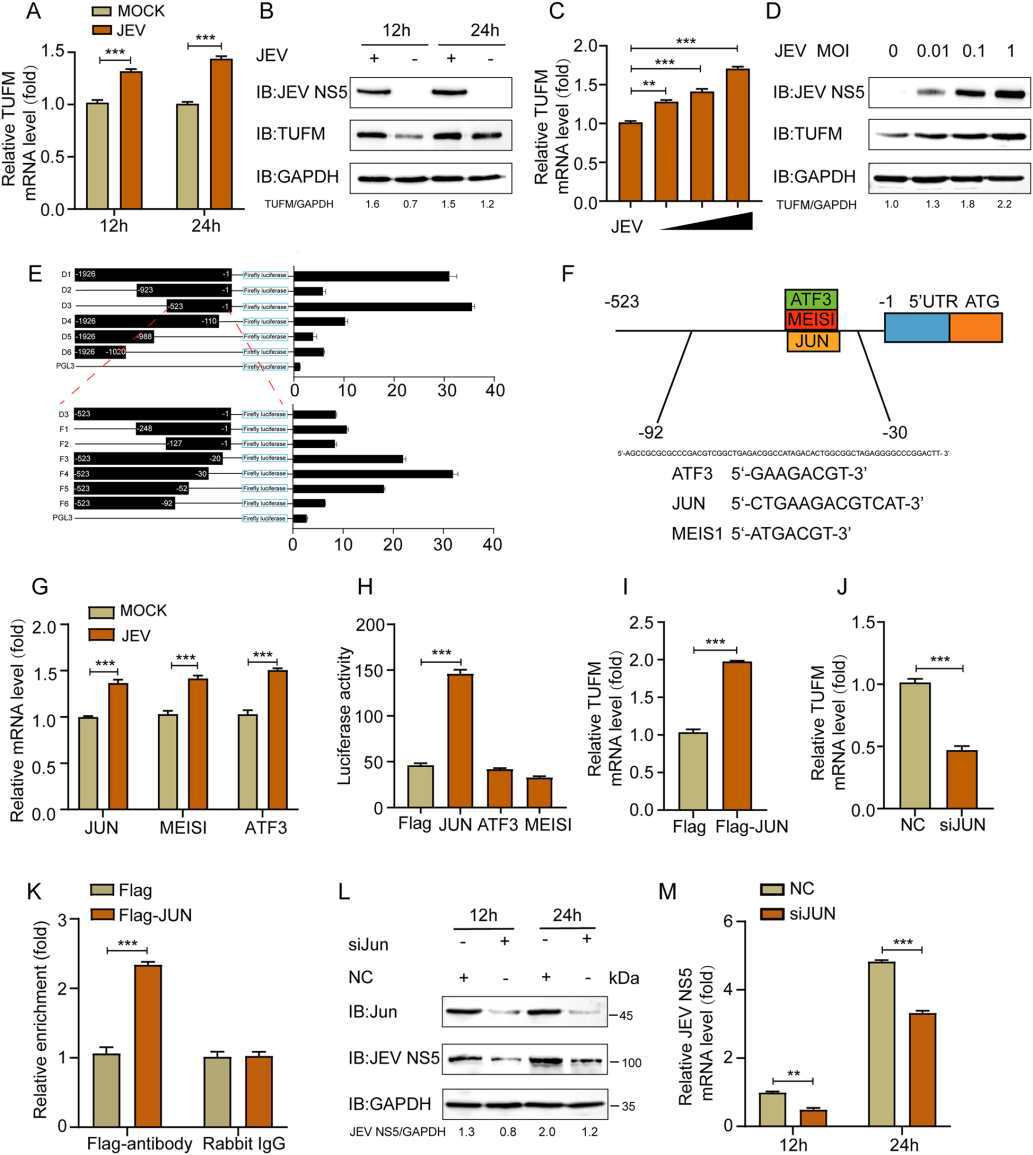
**JEV infection upregulates TUFM through JUN.** **A **and **B** Cells were infected with JEV (MOI = 0.5) and collected at the indicated time points. Western blotting and qRT-PCR were carried out to measure the endogenous TUFM expression. GAPDH was used as the internal control. **C** and **D** HEK293T cells infected with JEV at different MOIs were analyzed using western blotting and qRT-PCR. **E** HEK293T cells were transfected with the pGL3-Basic luciferase vector carrying truncated constructs (–1926 to –1) of the TUFM promoter and analyzed for luciferase activity. **F** Promoters of TUFM were predicted using JASPAR. **G** Relative mRNA levels of the predicted genes, in HEK293T cells infected with JEV, were determined using qRT-PCR. **H** Luciferase activity was determined in the cells overexpressing the predicted genes. **I** TUFM mRNA levels in JUN-overexpressing HEK293T cells were determined using qRT-PCR. **J** The mRNA expression of TUFM in siJUN-treated HEK293T cells was quantified by qRT-PCR. **K** Following transfection with Flag-JUN plasmid or vector, ChIP analysis for HEK293T cells subjected to harvesting and treatment. **L**, **M** HEK293T cells were transfected with si*JUN* or *siNC* and subsequently infected with JEV (MOI = 0.5), then we analyzed using western blotting and qRT-PCR.

Using iTRAQ proteomics, we identified the mitochondrial protein TUFM as a novel interaction partner of the JEV NS5 protein (Additional file1). To determine the effect of JEV infection on TUFM expression, HEK293T cells were infected with JEV (MOI = 0.5). We found that TUFM expression was significantly upregulated at both the protein and mRNA levels in infected cells (Figures 1A, B). JEV upregulated TUFM expression in a dose-dependent manner (Figures 1C, D), implying that TUFM may be actively regulated in response to JEV infection.

To identify the transcription factors regulating TUFM expression, we constructed a series of luciferase reporter plasmids containing the full-length or truncated TUFM promoter. Luciferase activity was then measured following transfection. The promoter constructs (–531 ~ –1) exhibited high luciferase activity, suggesting that the core promoter region is located within this region. To further refine the core promoter segments, the –531 ~ –1 fragment was truncated. Notably, comparison between the –531 ~ –1 and –531 ~ –92 constructs revealed a significant reduction in luciferase activity upon removal of the –92 ~ –30 region. Therefore, the probable location of the TUFM core promoter was identified as the –92 to –30 region (Figure 1E). We used the JASPAR database to predict transcription factors that potentially regulate TUFM expression. This analysis revealed that ATF3, JUN, and MEIS1 could bind to the core promoter region of TUFM (Figure 1F). Thereafter, the ATF3, JUN, and MEIS1 mRNA levels were assessed using qRT-PCR. ATF3, JUN, and MEIS1 were upregulated, which was consistent with the upregulation of TUFM during JEV infection (Figure 1G). We overexpressed ATF3, JUN, and MEIS1 and assessed their effects on the TUFM promoter. Among these, only JUN significantly upregulated TUFM-driven luciferase activity (Figure 1H). Overexpression of JUN led to increased mRNA levels of TUFM (Figure 1I). Conversely, knockdown of JUN in HEK293T cells led to a reduction in TUFM mRNA (Figure 1J). A chromatin immunoprecipitation (ChIP) assay using Flag-JUN was also done to immunoprecipitate the TUFM core promoter. JUN is directly bound to the TUFM promoter, regulating its expression, as shown in Figure 1K. To assess the level of JEV replication after JUN knockdown, we performed Western blotting and qPCR analyses, which revealed that JUN knockdown resulted in a marked reduction in both JEV protein expression and viral mRNA levels (Figures 1L, M). All of these findings imply that the transcription factor JUN can affect the expression of TUFM in JEV-infected cells.

### TUFM promoted JEV proliferation

**Figure 2:**
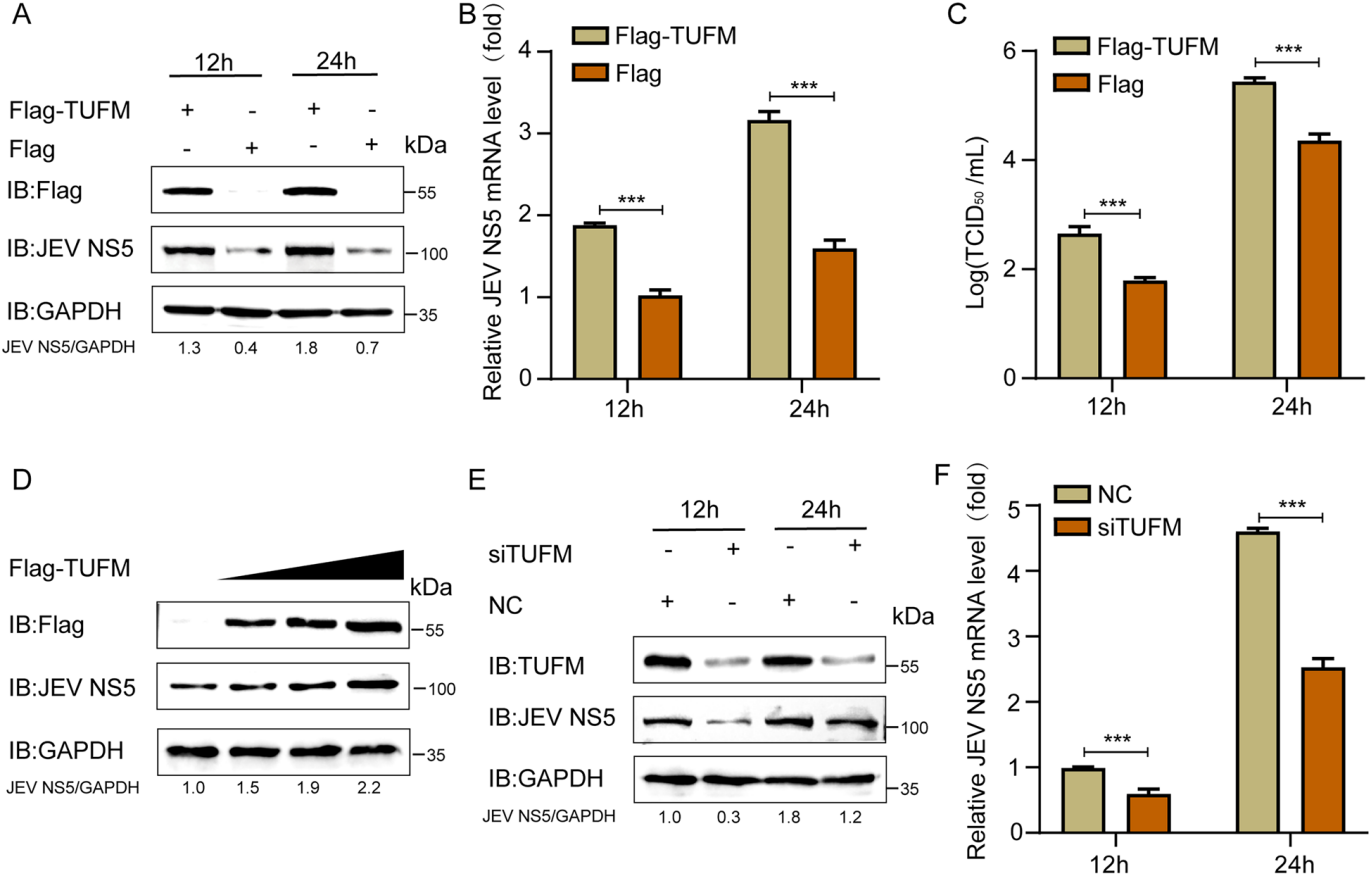
**TUFM promotes JEV infection.** **A**-**C** SH‑SY5Y cells were transfected with the Flag-TUFM plasmid and then infected with JEV at an MOI of 0.5. The cell lysates were harvested for western blotting, qRT-PCR, and TCID_50_ assays. **D** SH‑SY5Y cells were transfected with increasing doses of Flag-TUFM plasmids and then analyzed using western blotting. **E, F** SH‑SY5Y cells were transfected with si*TUFM* or *siNC* and subsequently infected with JEV (MOI = 0.5), then we analyzed using western blotting and qRT-PCR.

To investigate the role of TUFM in JEV infection, we performed gain- and loss-of-function studies in SH‑SY5Y cells. We transfected Flag‑TUFM into SH‑SY5Y cells and subsequently infected them with JEV (MOI = 0.5). Western blot analysis revealed that TUFM overexpression increased JEV NS5 levels in SH‑SY5Y cells (Figure 2A). Similarly, in the qRT-PCR analysis, the Flag-TUFM group presented higher JEV NS5 mRNA levels (Figure 2B). Consistently, viral titers in the supernatant were significantly higher in TUFM‑overexpressing cells than in vector‑transfected controls (Figure 2C). Moreover, TUFM promoted JEV proliferation in a dose-dependently manner (Figure 2D). Conversely, knockdown of TUFM in SH‑SY5Y cells inhibited JEV replication (Figure 2E, F). Collectively, these approaches demonstrate that TUFM is necessary to promote JEV replication, highlighting its critical role as a host factor facilitating viral propagation.

### TUFM acts as a negative regulator of the IFN-β signaling pathway.

**Figure 3:**
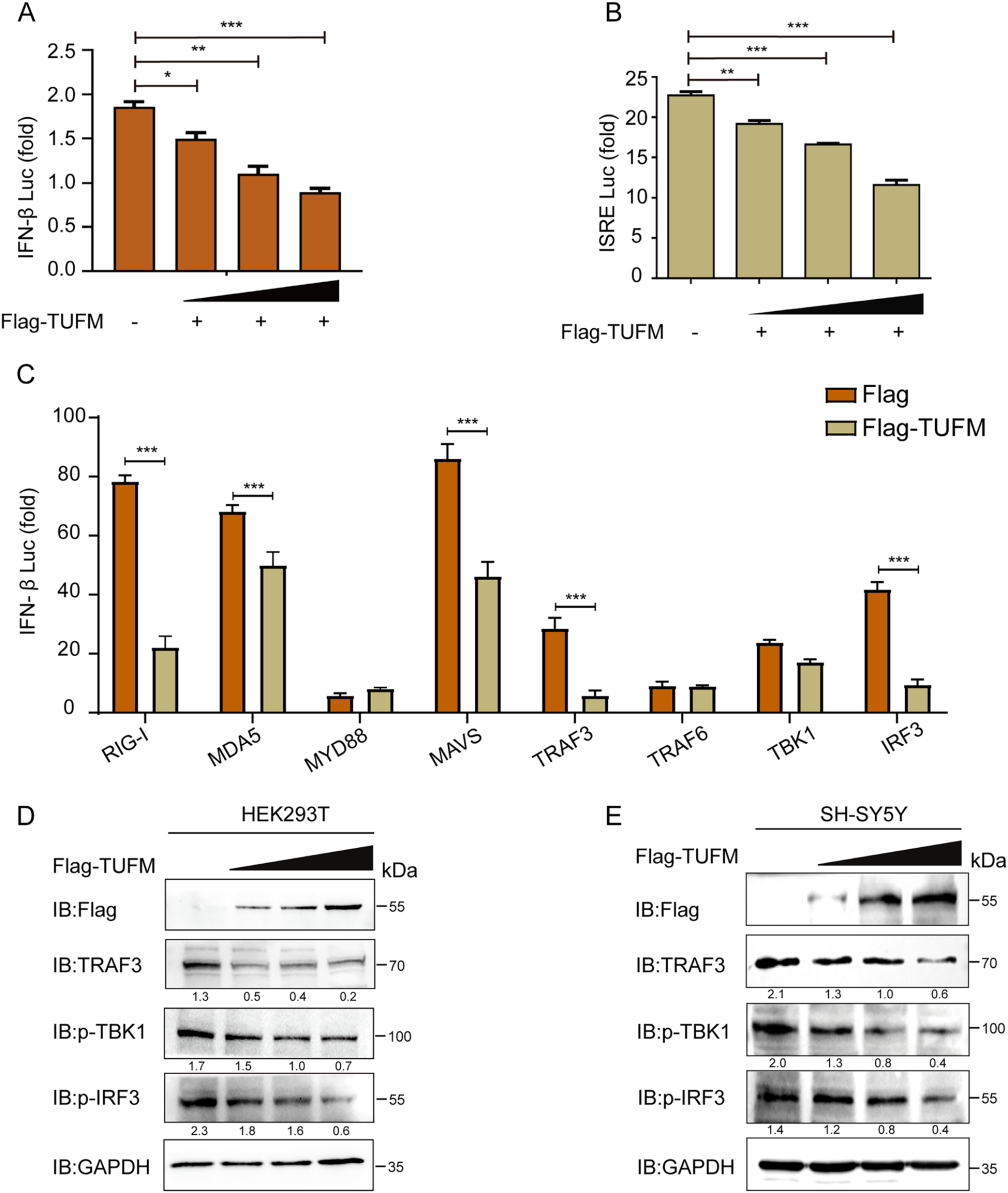
**TUFM inhibits IFN expression.** **A** and **B** HEK293T cells were co-transfected with pIFNβ-Luc or pISRE-Luc and pRL-TK along with increasing doses of Flag-TUFM and then harvested for the luciferase assay. **C** Flag-TUFM, pIFNβ-Luc, and the plasmids encoding RIG-I, MDA5, MYD88, MAVS, TRAF3, TRAF6, TBK1, or IRF3 were co-transfected into HEK293T cells followed by the luciferase assay. **D** HEK293T cells were transfected with increasing amounts of Flag-TUFM plasmids and then harvested for the western blot. **E** SH‑SY5Y cells were transfected with increasing amounts of Flag-TUFM plasmids and then harvested for the western blot.

The inherent immunity acts as the first line of defense against various pathogens [23–25]. However, the virus has developed an impressive variety of strategies to evade the host innate immunity [26, 27]. In this study, overexpression of TUFM in HEK293T cells inhibited ISRE and IFN-β promoter activity in a dose-dependent manner (Figures 3A, B). To elucidate how TUFM suppresses IFN signaling, we performed a luciferase reporter assay. It was found that TUFM could inhibited the IFN-β promoter activity through RIG-I, MDA5, MAVS, or TRAF3 (Figure 3C). Given that TRAF3 acts downstream of RIG‑I and MAVS in the IFN pathway, we hypothesized that TUFM‑mediated suppression occurs through targeting TRAF3. To investigate downstream signaling, we overexpressed Flag‑TUFM in HEK293T and SH‑SY5Y cells. Western blot analysis revealed that TUFM overexpression significantly reduced the protein levels of TRAF3, p‑TBK1, and p‑IRF3 (Figures 3D, E). Consistent with the central role of TRAF3 in the IFN‑β activation cascade, these results indicate that TUFM suppresses type I interferon signaling primarily through the degradation of TRAF3, which subsequently impairs the phosphorylation and activation of its downstream effectors, TBK1 and IRF3.

### TUFM induced the autophagic degradation of TRAF3

**Figure 4:**
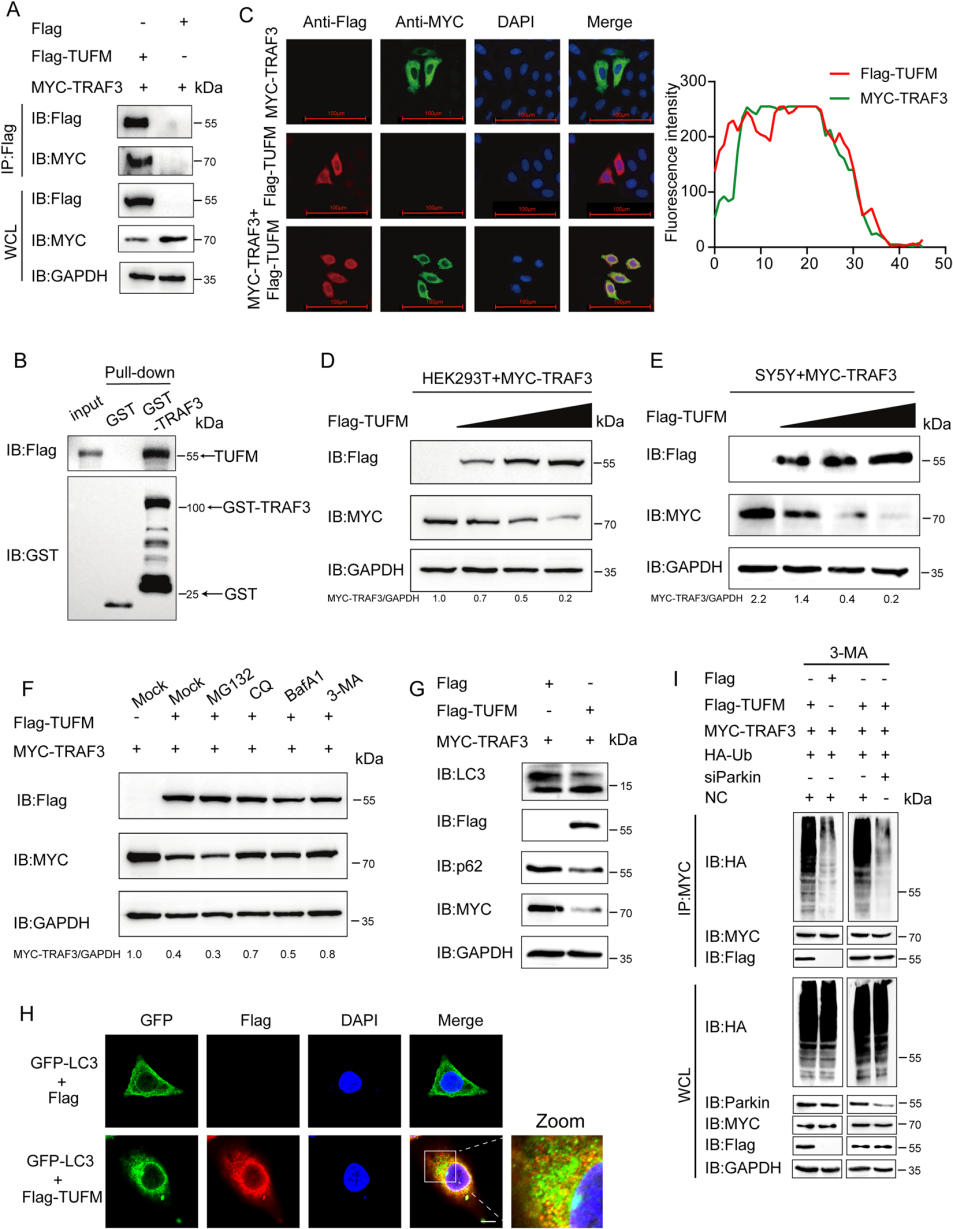
**TUFM degrades TRAF3 through selective autophagy. A** HEK293T cells were transfected with the Flag-TUFM and MYC-TRAF3 plasmids, and then the cells were processed for IP with anti-Flag magnetic beads. **B** A GST affinity-isolation assay was used to detect the interaction of GST-TRAF3 and TUFM. **C** Flag-TUFM and MYC-TRAF3 plasmids were transfected into Hela cells labeled with antibodies and then analyzed using confocal immunofluorescence microscopy. Scale bars: 100 µm. Quantitative Pearson's co-localization analysis was performed on the right panel. **D**, **E** MYC-TRAF3 and increasing amounts of Flag-TUFM plasmids were co-transfected into HEK293T and SH‑SY5Y cells, and the cellular lysates were analyzed using western blotting. **F** Flag-TUFM and MYC-TRAF3 plasmids were co-transfected into HEK293T cells, which were then treated with MG132, CQ, BafA1, and 3-MA. Western blotting was utilized to detect the protein in the cell lysates. **G** HEK293T cells were transfected with Flag-TUFM and MYC-TRAF3. At 24 hpt, the cells were harvested for western blot analysis. **H** HeLa cells were transfected with GFP-LC3 along with empty vector or Flag-TUFM. At 24 hpt, cells were analysis by confocal microscopy (scale bar: 100 μm). **I** HEK293T cells were co-transfected with plasmids expressing MYC-TRAF3, HA-ubiquitin (HA-Ub), and either Flag-TUFM or the empty Flag vector as indicated. Where specified, cells were also transfected with Parkin-specific siRNA (siParkin) or a non-targeting control siRNA (NC). At 24 hpt, whole-cell lysates were subjected to immunoprecipitation with an anti-MYC antibody, followed by immunoblotting with the indicated antibodies.

To elucidate how TUFM mediates TRAF3 degradation, we examined their interaction by co‑immunoprecipitation (Co‑IP) and GST pull‑down assays. The results revealed that TUFM interacted with TRAF3 (Figures 4A, B). The confocal immunofluorescence assay revealed that TRAF3 colocalized with TUFM in the cytoplasm (Figure 4C). The right panel shows the analysis of colocalization between TUFM and TRAF3. Co‑transfection of TRAF3 with TUFM (but not with empty vector) in HEK293T and SH‑SY5Y cells reduced TRAF3 protein levels, indicating that TUFM promotes TRAF3 degradation (Figures 4D, E). In eukaryotic cells, the ubiquitin–proteasome and autophagy-lysosome systems constitute the main protein degradation pathways [28]. Therefore, to identify which of these two pathways is involved in TUFM-initiated TRAF3 degradation, TRAF3 and TUFM were ectopically expressed in HEK293T cells, followed by treatment with the autophagy inhibitors baffamycin A1 (BafA1), 3-methyladenine (3-MA), and chloroquine (CQ), or a proteasome inhibitor (MG132). Western blot analysis revealed that TRAF3 expression was rescued in the cells treated with BafA1, CQ, and 3-MA but not in the MG132-treated cells (Figure 4F). Furthermore, the endogenous LC3-I to LC3-II conversion (Figure 4G) and LC3-GFP puncta formation (Figure 4H) were significantly increased in HEK293T cells that transfected Flag-TUFM. TUFM expression, but not the empty vector control, readily induced TRAF3 ubiquitination upon co‑transfection of wild‑type ubiquitin and TRAF3 in HEK293T cells. Notably, this TUFM‑induced ubiquitination was abolished when Parkin was knocked down (Figure 4I). Altogether, these results demonstrate that TUFM targets TRAF3 for degradation via the autophagy pathway.

### JEV NS5 cooperates with TUFM to target TRAF3 for degradation through the Parkin‑p62 autophagy pathway

**Figure 5:**
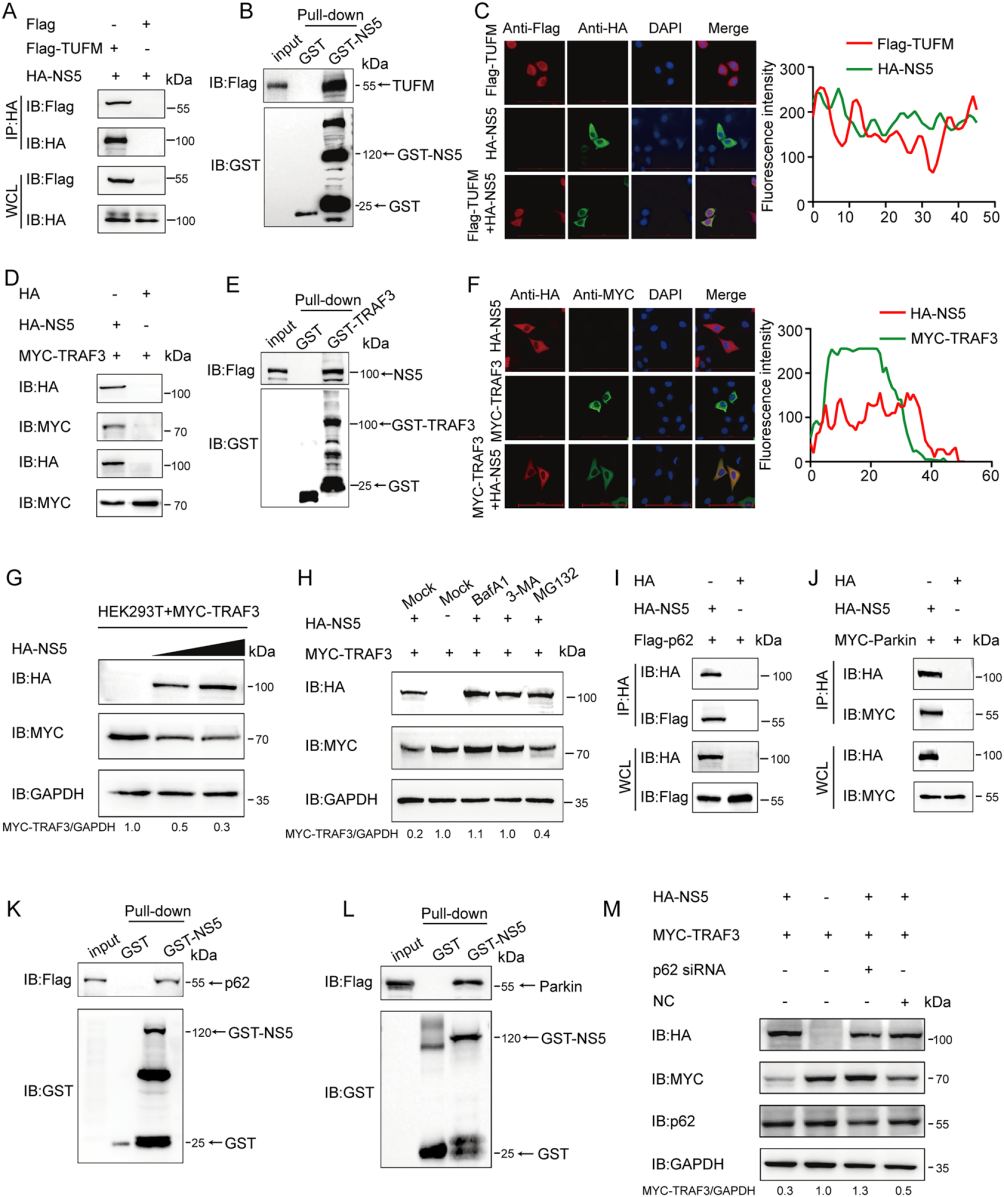
**NS5 degrades TRAF3 by interacting with TUFM. A** HEK293T cells were transfected with Flag-TUFM- and HA-NS5-encoding plasmids. Co-IP analysis was used to analyze the precipitated proteins. **B** The GST affinity-isolation assay was used to detect GST-NS5 and TUFM. **C** Flag-TUFM and HA-NS5 plasmids were transfected into HeLa cells, which were then analyzed using confocal immunofluorescence microscopy. Scale bars: 100 µm. Quantitative Pearson's co-localization analysis was performed on the right panel. **D** HEK293T cells were transfected with MYC-TRAF3-encoding and HA-NS5-encoding plasmids. Co-IP analysis was used to analyze the precipitated proteins. **E** A GST affinity isolation assay was used to detect GST-NS5 and TRAF3. **F** MYC-TRAF3 and HA-NS5 plasmids were transfected into HeLa cells, and the cells were labeled with antibodies and then analyzed using confocal immunofluorescence microscopy. Scale bars: 100 µm. Quantitative Pearson's co-localization analysis was performed on the right panel. **G** HEK293T cells were co-transfected with the MYC-TRAF3 and HA-NS5 plasmids, and the expression of TRAF3 was determined using western blotting. **H** HA-NS5 and MYC-TRAF3 plasmids were co-transfected into HEK293T cells, after which the cells were treated with MG132, CQ, BafA1, or 3-MA. Western blotting was utilized to detect the protein in the cell lysates. **I** and **J** HEK293T cells were co-transfected with HA-NS5 and Flag-p62 or MYC-Parkin plasmids for Co-IP. **K**, **L** The GST affinity isolation assay was used to detect GST-NS5 and p62 or Parkin. **M** HEK293T cells were transfected with HA-NS5, MYC-TRAF3 plasmids, or sip62 RNA. Western blot analysis was conducted to determine the protein levels of TRAF3.

Using the iTRAQ technique, it was demonstrated that TUFM may be involved in the interaction with JEV NS5 (see Additional file 1). Therefore, we employed co-immunoprecipitation (Co-IP), GST affinity isolation, and confocal microscopy assays to verify that TUFM interacted with NS5 (Figures 5A, B), and that the two proteins were colocalized in the cytoplasm (Figure 5C). The right panel shows the analysis of colocalization between TUFM and NS5. The above results suggested that the host factor TUFM could degrade TRAF3 to downregulate IFN expression. Because NS5 is involved mainly in the regulation of interferon [11] and TUFM was demonstrated to interact with JEV NS5, it was speculated that NS5 and TUFM can work together to degrade TRAF3. As shown in Figures 5D-F, NS5 interacted and colocalized with TRAF3. Right panel, shows the colocalization analysis of TUFM and NS5. To determine the effect of NS5 on TRAF3, we co‑transfected HA‑NS5 and MYC‑TRAF3 plasmids in HEK293T cells. We found that NS5 overexpression led to a dose‑dependent downregulation of TRAF3 protein. (Figure 5G). Next, using a degradation inhibitor BafA1, 3-MA, MG132, it was demonstrated that the NS5 protein could degrade TRAF3 through autophagic degradation (Figure 5H). In selective autophagy, substrates are ubiquitinated by specific E3 ubiquitin ligases, which enables their recognition by cargo receptors and subsequent delivery to autophagosomes for selective degradation [28]. Based on our iTRAQ results, p62 was identified as a potential autophagy receptor. The study first explicitly proposed the molecular mechanism by which p62 targets damaged mitochondria to autophagosomes through binding to Parkin-mediated ubiquitin chains. Therefore, we employed Co-IP and GST pull-down assays and then revealed that NS5 interacted with p62 and Parkin (Figures 5I-L). In order to investigate the degradation of TRAF3 protein by NS5 through p62, we co-transfected HA-NS5 plasmids, MYC-TRAF3, and p62 siRNA into HEK293T cells. According to western blot results, NS5-triggered degradation of TRAF3 was suppressed through knockdown p62 (Figure 5M). These results suggested that JEV NS5 could degrade TRAF3 through autophagy via the Parkin-p62 autophagosome pathway.

### TRAF3 protein was degraded through the TUFM-Parkin-p62-autophagosome pathway

**Figure 6:**
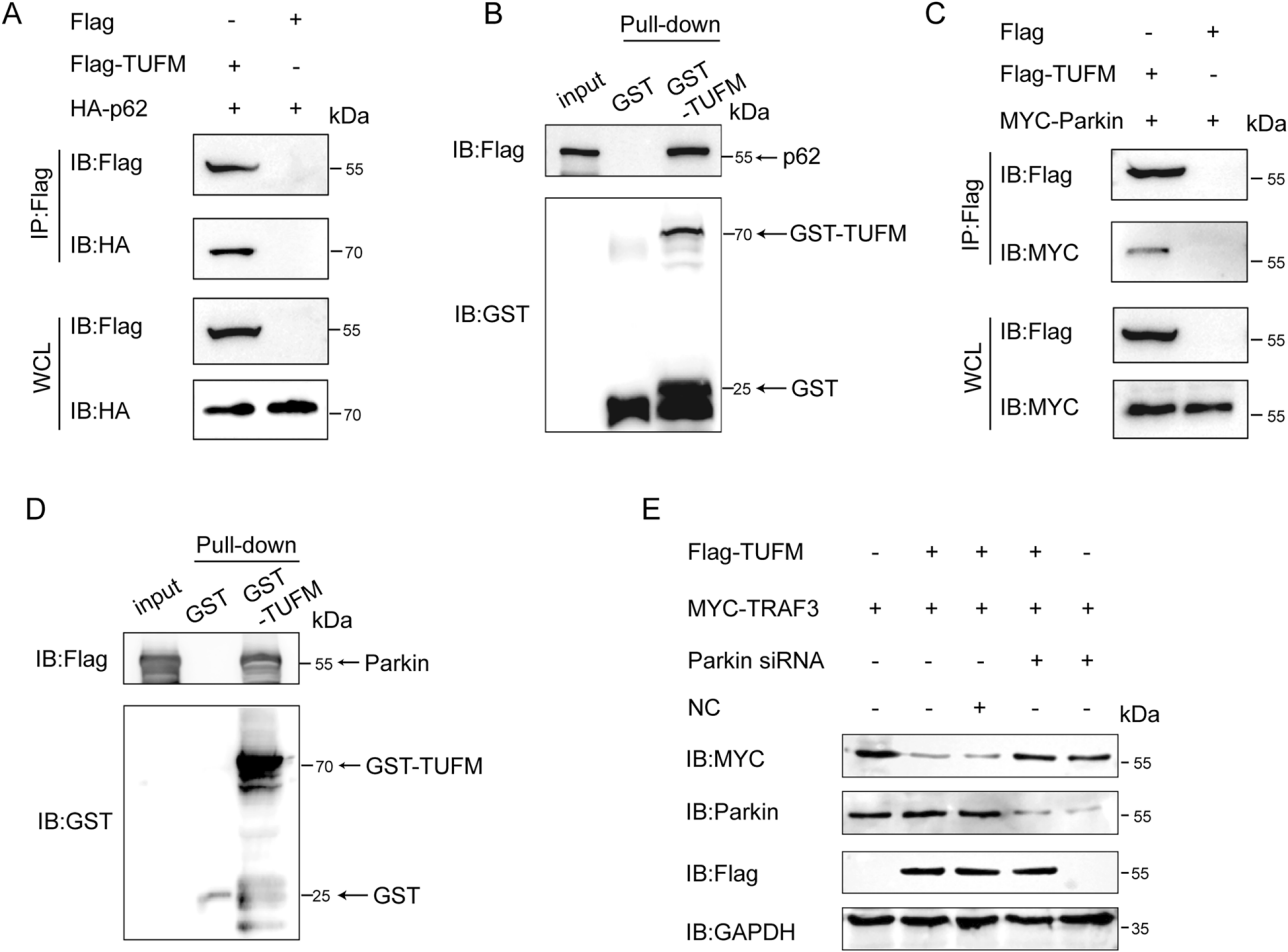
**TUFM degrades TRAF3 through the Parkin-p62-autophagosome pathway.** **A** HEK293T cells were treated with Flag-TUFM- and HA-p62-encoding plasmids, and Co-IP assay was used to analyze the precipitated proteins. **B** GST pull-down assays were used to analyze the interaction between GST-TUFM and HA-p62. **C** HEK293T cells were treated with Flag-TUFM and MYC-Parkin-encoding plasmids, and Co-IP assay was used to analyze the precipitated proteins. **D** The GST affinity-isolation assay was used to detect the interaction of GST-TUFM and Parkin. **E** HEK293T cells were co-transfected with MYC-TRAF3, Flag-TUFM plasmids, and siParkin, and the cells were analyzed using western blotting.

This study revealed that TUFM interacted with p62 through Co-IP (Figure 6A). In addition, we also performed GST pull‑down assays, confirming the direct interaction between TUFM and p62 (Figure 6B). To verify that ubiquitin ligases were involved in the ubiquitination of TRAF3, HEK293T cells were co-transfected with Flag-TUFM and MYC-Parkin plasmids. According to Co-IP assay results, TUFM could bind to Parkin (Figure 6C). We also confirmed the direct interaction between TUFM and Parkin by GST pull-down assay. (Figure 6D), suggesting that TUFM might have degraded TRAF3 through selective autophagy involving p62 and Parkin. Western blot analysis confirmed that Parkin knockdown prevented TUFM‑mediated TRAF3 degradation, indicating that Parkin is essential for this process (Figure 6E). Taken together, these data demonstrated that the cargo receptor p62 and the ubiquitination enzyme Parkin mediate the TUFM-induced TRAF3 degradation.

## Discussion

JEV, as one of the most common insect-borne diseases of human central nervous system, is considered a major public health threat worldwide [29]. Recent studies have shown that the interplay between JEV protein and host proteins has important effects on viral replication, natural immune evasion, and the induction of host protective immunity [30]. According to the results, JEV infection effectively upregulated TUFM expression. In order to further investigate the transcriptional regulators of TUFM expression, we amplified the TUFM promoter sequence, and it was discovered that the minimum TUFM core promoter was located between -92 and -30 positions. During JEV infection, multiple signaling pathways are widely activated, resulting in synchronized upregulation of multiple transcription factors such as ATF3, JUN, and MEIS1 at the transcriptional level. However, elevated transcription factor mRNA levels do not necessarily mean that it can directly activate the promoter of a particular target gene, so we conducted dual luciferase and chromatin immunoprecipitation (ChIP) assays, which collectively demonstrated that JUN can bind to the TUFM promoter region and significantly upregulate its luciferase activity.

**Figure 7:**
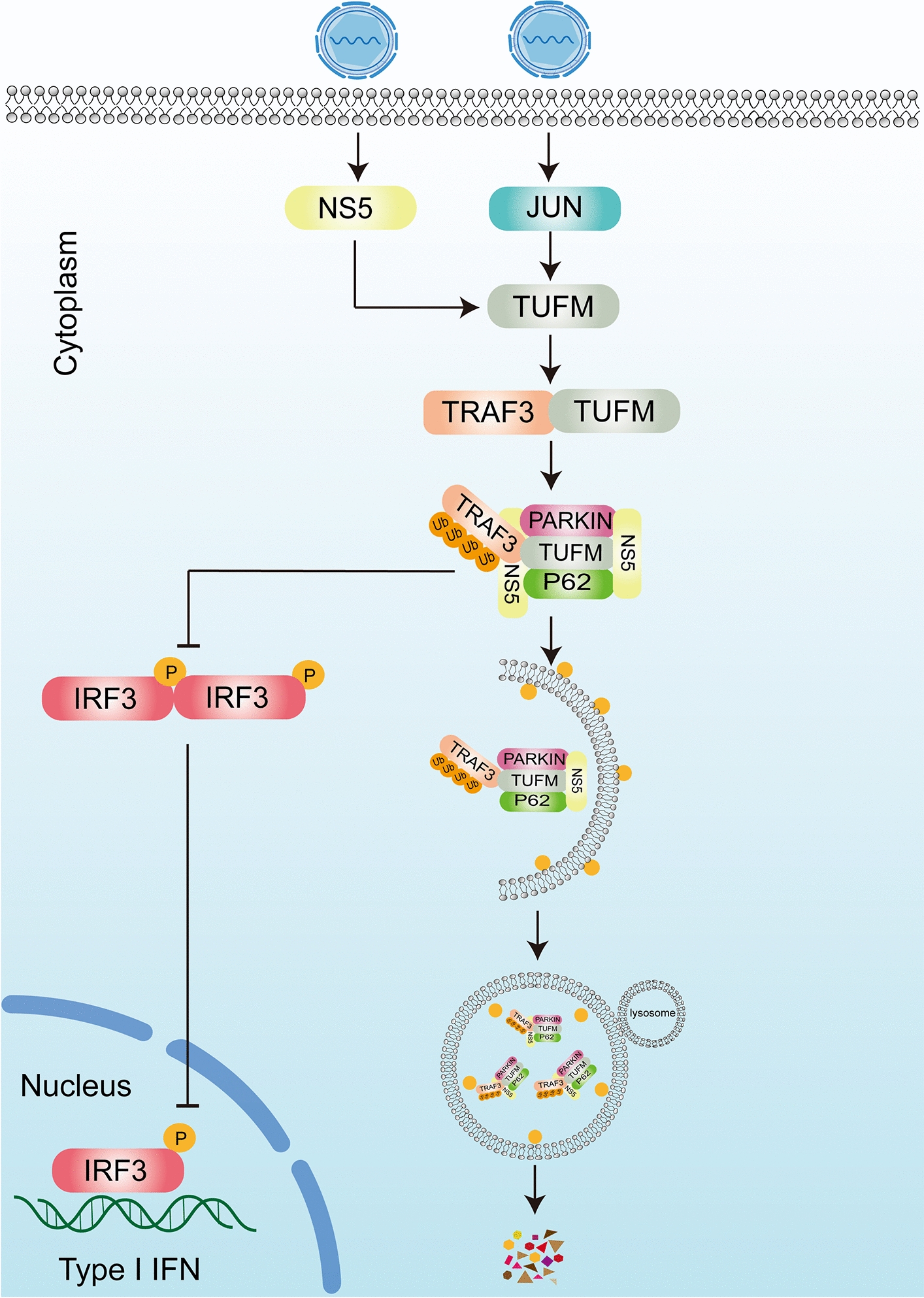
**NS5 and TUFM degrade TRAF3 to suppress IFN-β and enhance JEV replication.** JEV can use the NS5 protein and host TUFM protein to inhibit IFN production by degrading TRAF3 through the autophagy pathway. Both the NS5 protein and the host TUFM protein interact with p62 and Parkin to inhibit IFN production by degrading TRAF3 through autophagic degradation to promote JEV proliferation.

The mitochondrial protein TUFM is a crucial multifunctional molecule [31], and previous studies have revealed its interaction with Atg5-Atg12 and its suppressive effect on the RIG-I-mediated secretion of type I IFN [32]. Following mitochondrial translocation and interaction with TUFM, both the HPIV3M and Hantavirus (HTNV) Gn proteins recruit LC3B to mitochondria. There, they act as autophagy receptors to trigger mitophagy, thereby suppressing the type I interferon response [33, 34]. The SVA (Senecavirus A) 2C protein mediates mitophagy by interplaying directly with TUFM and induces mitophagy to promote SVA replication [35]. Previous studies have shown that TUFM associates with bcNLRX1, thereby suppressing MAVS‑driven interferon induction. Our study demonstrates that TUFM promotes JEV replication by degrading TRAF3, thereby suppressing type I interferon production and facilitating efficient viral propagation (Figure 7). Eukaryotic cells primarily degrade proteins via two major pathways: the ubiquitin–proteasome pathway and autophagy pathways [36]. Autophagy begins with the formation of autophagosomes, which are responsible for the engulfment of damaged components, and ends with the fusion of lysosomes and autophagosomes [37]. This study revealed that TUFM can reduce the protein levels of TRAF3. TUFM is capable of inducing the autophagic degradation of TRAF3, and we have demonstrated a direct interaction between Parkin and TRAF3. Therefore, we propose that Parkin is a candidate E3 ligase involved in TRAF3 regulation.

JEV non‑structural proteins antagonize the interferon pathway, disrupting its signaling functions to enable immune evasion [38, 39]. In particular, the non-structural protein NS1 of JEV inhibits the production of type I interferons by targeting the mitochondrial antiviral signaling protein (MAVS) [40]. The non-structural protein NS4B of JEV can inhibit the production of IFN-β by interfering with the TLR3-TRIF pathway [41].This study establishes a novel TUFM-NS5-TRAF3 axis through which JEV suppresses host interferon responses. We demonstrate that the viral NS5 protein cooperates with mitochondrial TUFM to recruit Parkin and p62, leading to autophagic degradation of the immune adaptor TRAF3. Using Co-IP, GST pull-down, Confocal and reporter assays, we validated the molecular interactions and functional outcomes of this pathway. These results provide insights into a previously unrecognized viral immune evasion mechanism and identify molecules that may serve as potential points for intervention against JEV.

## Significance

Epidemic encephalitis is a JEV-induced acute central nervous system disease that is contagious. During JEV infection, the virus may regulate the host antiviral reaction to promote viral replication.This research unraveled that TUFM recruits the E3 ubiquitin ligase Parkin and autophagic receptor p62 to form a ubiquitin-protein complex that targets TRAF3 degradation. In addition, it was revealed that JEV uses the viral NS5 protein to degrade TRAF3 through selective autophagy to inhibit IFN-β production and promote JEV proliferation. These findings provided insights into novel routes through which JEV evades inherent immunity in the host, thereby improving the understanding of JEV pathogenesis.

## Supplementary Information

**Additional file** **1**. **iTRAQ proteomics data of pCMV-HA-NS5 overexpression.** Quantitative proteomic analysis of cells expressing pCMV-HA-NS5 versus pCMV-HA control.

## Data Availability

The data that support the findings of this study are available from the corresponding author upon reasonable request.
